# The impact of CLDN18.2 expression on effector cells mediating antibody-dependent cellular cytotoxicity in gastric cancer

**DOI:** 10.1038/s41598-024-68970-y

**Published:** 2024-08-02

**Authors:** Akira Matsuishi, Shotaro Nakajima, Motonobu Saito, Katsuharu Saito, Satoshi Fukai, Hideaki Tsumuraya, Ryo Kanoda, Tomohiro Kikuchi, Azuma Nirei, Akinao Kaneta, Hirokazu Okayama, Kosaku Mimura, Hiroyuki Hanayama, Wataru Sakamoto, Tomoyuki Momma, Zenichiro Saze, Koji Kono

**Affiliations:** 1 Department of Gastrointestinal Tract Surgery, Fukushima Medical University School of Medicine, Fukushima, Japan; 2 Department of Multidisciplinary Treatment of Cancer and Regional Medical Support, Fukushima Medical University School of Medicine, 1 Hikariga-oka, Fukushima City, Fukushima 960-1295 Japan; 3 Department of Blood Transfusion and Transplantation Immunology, Fukushima Medical University School of Medicine, Fukushima, Japan

**Keywords:** Gastric cancer, CLDN18.2, NK cells, Monocytes/macrophages, Gastric cancer, Tumour immunology

## Abstract

Activating antibody-dependent cellular cytotoxicity (ADCC) by targeting claudin-18 isoform 2 (CLDN18.2) using zolbetuximab, a monoclonal antibody against CLDN18.2, has been considered a promising novel therapeutic strategy for gastric cancer (GC). However, the impact of CLDN18.2 expression on natural killer (NK) cells and monocytes/macrophages—crucial effector cells of ADCC—in GC has not been fully investigated. In the present study, we assessed the impact of CLDN18.2 expression on clinical outcomes, molecular features, and the frequencies of tumor-infiltrating NK cells and macrophages, as well as peripheral blood NK cells and monocytes, in GC by analyzing our own GC cohorts. The expression of CLDN18.2 did not significantly impact clinical outcomes of GC patients, while it was significantly and positively associated with Epstein–Barr virus (EBV) status and PD-L1 expression. The frequencies of tumor-infiltrating NK cells and macrophages, as well as peripheral blood NK cells and monocytes, were comparable between CLDN18.2-positive and CLDN18.2-negative GCs. Importantly, both CLDN18.2 expression and the number of tumor-infiltrating NK cells were significantly higher in EBV-associated GC compared to other molecular subtypes. Our findings support the effectiveness of zolbetuximab in CLDN18.2-positive GC, and offer a novel insight into the treatment of this cancer type, highlighting its potential effectiveness for CLDN18.2-positive/EBV-associated GC.

## Introduction

Gastric cancer (GC) is the third most common cause of cancer-related deaths worldwide^1,2^. Despite advancements in treatment options based on the classifications of GC according to molecular features, the prognosis of GC remains poor, and novel treatment options are needed. Claudin-18 isoform 2 (CLDN18.2) is a tight junction protein normally and specifically expressed in gastric mucosa cells^3^. The expression of CLDN18.2 is sustained during the malignant transformation of gastric mucosa, and its epitopes become exposed on the surface of GC cells due to the loss of cell polarity. Therefore, CLDN18.2 is considered a promising therapeutic target for GC^4,5^. In recent phase 3 clinical trials, including the SPOTLIGHT study (NCT03504397)^6^ and GLOW study (NCT03653507)^7^, first-line zolbetuximab, a chimeric monoclonal antibody against CLDN18.2, combined with cytotoxic chemotherapy has been demonstrated to result in significant clinical improvement in patients with CLDN18.2-positive, HER2-negative, locally advanced unresectable or metastatic gastric or gastro-oesophageal junction adenocarcinoma. Although the anti-CLDN18.2 antibody mediates tumor cell death through antibody-dependent cellular cytotoxicity (ADCC) triggered by immune effector cells, including natural killer (NK) cells and macrophages^8^, the impact of CLDN18.2 expression on the frequency of tumor-infiltrating NK cells and macrophages, as well as peripheral blood NK cells and monocytes in patients with GC, is not yet fully understood.

ADCC is triggered via immune effector cells expressing Fc-γ receptors (FcγRs), and FcγRIIIA, also know as CD16a, is essential for NK cell-mediated ADCC^9^. Human NK cells can be broadly categorized into two major subsets: CD56^dim^CD16^+^ and CD56^bright^CD16^−^ NK cells, distinguished by their expression of CD56 and CD16^10^. The former subset accounts for approximately 90% of NK cells, displays a high level of perforin and plays a crucial role in cytotoxicity, whereas CD56^bright^CD16^−^ NK cells have a minor role in cytotoxicity but predominantly produce cytokines^10^. Our previous study, as well as others, demonstrated that the frequency and cytotoxicity of tumor-infiltrating NK cells, particularly CD56^dim^CD16^+^ NK cells, progressively decrease in GC patients as the disease advances^11,12^. Li et al*.* reported that decreased infiltration of NK cells into intratumoral regions was significantly associated with decreased survival and disease progression in GC patients^13^. Additionally, Wang et al*.* found that tumor formation might negatively impact the frequency and activity of blood NK cells in GC patients^14^.

Several subtypes of macrophages and subsets of monocytes also express CD16 to moderate extents, contributing to ADCC activity. Concerning tumor-associated macrophages (TAMs), two major subtypes exist: pro-inflammatory M1 TAMs and anti-inflammatory M2 TAMs^15^. Although CD16 might be expressed in both M1 and M2 phenotypes^16–19^, van Ravenswaay Claasen et al*.* reported that CD16^+^ macrophages may be involved in antitumor cytotoxicity^20^. Additionally, Zhu et al*.* found that the density of CD16^+^ TAMs significantly correlates with survival time in patients with GC^21^. On the other hand, human blood monocytes are classified into three subsets based on their expression of CD14 and CD16: classical (CD14^++^CD16^–^), intermediate (CD14^++^CD16^+^), and non-classical monocytes (CD14^+^CD16^++^)^22^. Yeap et al*.* reported that CD16^+^ monocytes exhibit ADCC activities, with non-classical monocytes showing particularly higher ADCC activity compared to that induced by intermediate monocytes^23^. Intriguingly, Eljaszewicz et al*.* reported a significant increase in the frequency of CD16^+^ intermediate and non-classical monocytes in patients with GC compared to healthy volunteers^24^. Furthermore, Jeong et al*.* reported that the frequency of tumor-infiltrating non-classical monocytes increased in GC patients with disease progression, although a high frequency of their infiltration into tumor tissues was associated with lower overall survival (OS)^25^. Thus, the subtypes of monocytes/macrophages remain controversial from the perspective of ADCC capability in GC.

In the present study, we investigated the association between CLDN18.2 expression and clinical outcomes, molecular features, or the number of tumor-infiltrating NK cells and macrophages in patients with GC using two cohorts: Fukushima Medical University (FMU) cohort 1 (*n* = 284) and 2 (*n* = 15). Additionally, we examined the correlation between CLDN18.2 expression in tumor tissues and the peripheral frequencies of blood NK cells and monocytes in patients with GC, analyzing FMU cohort 3 (*n* = 79). Furthermore, we validated our findings by analyzing public datasets of GC samples from The Cancer Genome Atlas (TCGA) and Gene Expression Omnibus (GEO).

## Results

### Impact of CLDN18.2 expression on clinicopathological features and prognosis in GC patients

**Figure 1:**
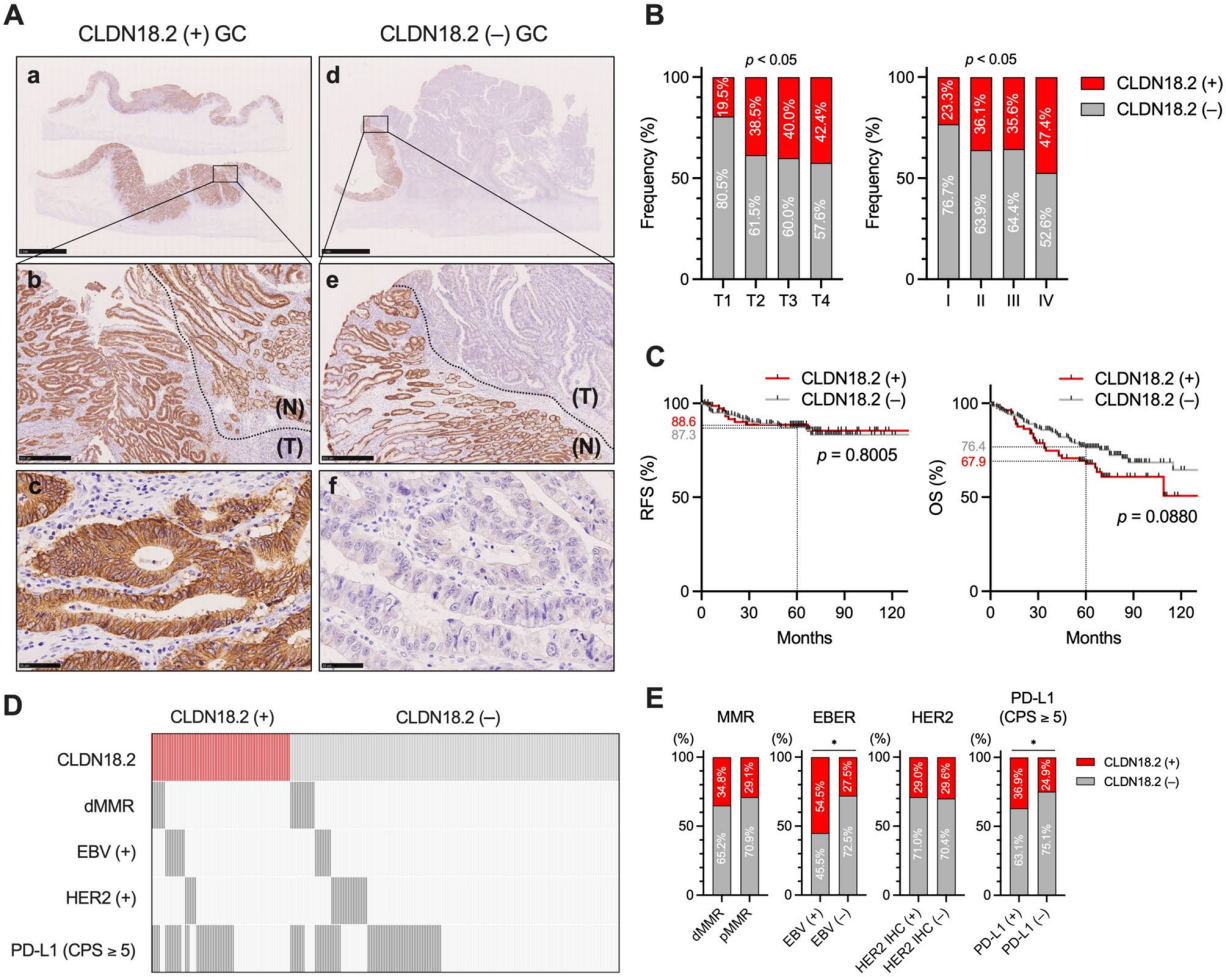
Association of CLDN18.2 expression with clinicopathological features and clinical outcomes in GC (FMU cohort 1). (**A**) Representative IHC images for CLDN18.2 in CLDN18.2 ( +) and CLDN18.2 (–) GCs in FMU cohort 1 (*n* = 284). Scale bars: 5 mm for **a** and **d**, 500 μm for **b** and **e**, and 50 μm for **c** and **f**. **N** denotes the adjacent normal region, while **T** denotes the tumor region. (**B**) Associations between CLDN18.2 expression detected by IHC and the depth of tumor invasion (T1, *n* = 149; T2, *n* = 39; T3, *n* = 30; T4, *n* = 66) or pathological TNM stage (I, *n* = 159; II, *n* = 61; III, *n* = 45; IV, *n* = 19) in patients with GC. (**C**) Kaplan–Meier curves for RFS and OS according to CLDN18.2 expression detected by IHC in patients with GC [CLDN18.2 ( +), *n* = 84; CLDN18.2 (–), *n* = 200]. (**D**) A panel illustrating the association between CLDN18.2 expression and molecular characteristics detected by IHC or in situ hybridization (for EBV status) in GC (*n* = 284). Dark grey indicates positive cases, and light grey indicates negative cases for dMMR, EBV, HER2, and PD-L1 (CPS ≥ 5). dMMR stands for deficient mismatch repair, EBV for Epstein-Barr virus, HER2 for human epidermal growth factor receptor 2, PD-L1 for programmed cell death ligand 1, and CPS for a combined positive score. (**E**) Associations between CLDN18.2 expression and molecular characteristics detected by IHC or in situ hybridization (for EBV status) in GC [dMMR, *n* = 23 vs pMMR, *n* = 261; EBV ( +), *n* = 22 vs EBV (–), *n* = 262; HER2 IHC ( +), *n* = 31 vs HER2 IHC (–), *n* = 253; PD-L1 ( +), *n* = 111 vs PD-L1 (–), *n* = 173]. Statistical significance was determined by Fisher’s exact test or the Chi-square test (**B**,**E**) and the log-rank test (**C**). *p < 0.05.

We initially assessed the association between CLDN18.2 status and clinicopathological features, as well as prognosis in GC patients. A total of 284 GC patients who had not received any neoadjuvant therapies were included in this analysis (FMU cohort 1). Among these, 84 cases (29.6%) were identified as CLDN18.2-positive [CLDN18.2 ( +)] (Table 1). Representative immunohistochemistry (IHC) images of CLDN18.2 ( +) and CLDN18.2-negative [CLDN18.2 (–)] cases are presented in Fig. 1A. CLDN18.2 expression significantly correlated with the depth of tumor invasion (*p* = 0.0014) and pathological TNM (pTNM) stage (*p* = 0.0475) in GC (Table 1, Fig. 1B). However, there were no statistically significant correlations between CLDN18.2 status and recurrence-free survival (RFS) (*p* = 0.8005, hazard ratio = 0.9067, 95% CI 0.4303–1.910) or OS (*p* = 0.0880, hazard ratio = 1.481, 95% CI 0.9077–2.416) (Fig. 1C). We also investigated the association of CLDN18.2 expression and the molecular features of GC, including the statuses of mismatch repair (MMR), Epstein-Barr virus (EBV), human epidermal growth factor receptor 2 (HER2), and programmed cell death ligand 1 (PD-L1) (Fig. 1D). Significant positive associations were observed between CLDN18.2 expresson and EBV status (*p* = 0.0131) or PD-L1 expression [combined positive score (CPS) ≥ 5] (*p* = 0.0334) (Table 1 and Fig. 1E).

**Table 1:** Clinicopathological characteristics of gastric cancer patients according to CLDN18.2 status (FMU cohort 1).

	Total	CLDN18.2 (–)	CLDN18.2 ( +)	*p*-value
	*n* = 284	*n* = 200 (70.4%)	*n* = 84 (29.6%)	
Age				0.829
Mean ± SD	68.0 ± 8.8	68.1 ± 9.0	67.8 ± 8.3	
Gender				0.1526
Male	203 (71.5%)	148 (74.0%)	55 (65.5%)	
Female	81 (28.5%)	52 (26.0%)	29 (34.5%)	
Location				0.8257
Upper	86 (30.3%)	62 (31.0%)	24 (28.6%)	
Middle	100 (35.2%)	74 (37.0%)	26 (31.0%)	
Low	83 (29.2%)	58 (29.0%)	25 (29.7%)	
Remnant GC	15 (5.3%)	6 (3.0%)	9 (10.7%)	
Histological type				0.3586
Differentiated	151 (53.2%)	103 (51.5%)	48 (57.1%)	
Undifferentiated	128 (45.1%)	94 (47.0%)	34 (40.5%)	
Unclear	5 (1.7%)	3 (1.5%)	2 (2.4%)	
Tumor invasion				0.0014
T1	149 (52.5%)	120 (60.0%)	29 (34.5%)	
T2	39 (13.7%)	24(12.0%)	15 (17.9%)	
T3	30 (10.6%)	18 (9.0%)	12 (14.3%)	
T4	66 (23.2%)	38 (19.0%)	28 (33.3%)	
Lymph node metastasis				0.3427
Absent	180 (63.4%)	131 (65.5%)	49 (58.3%)	
Present	103 (36.3%)	69 (34.5%)	34 (40.5%)	
Not available	1 (0.3%)	0 (0%)	1 (1.2%)	
Distant metastasis				0.3134
Absent	264 (93.0%)	188 (94.0%)	76 (90.5%)	
Present	20 (7.0%)	12 (6.0%)	8 (9.5%)	
pTNM stage				0.0475
I	159 (56.0%)	122 (61.0%)	37 (44.0%)	
II	61 (21.5%)	39 (19.5%)	22 (26.2%)	
III	45 (15.8%)	29 (14.5%)	16 (19.0%)	
IV	19 (6.7%)	10 (5.0%)	9 (10.7%)	
MMR				0.6346
Deficient	23 (8.1%)	15 (7.5%)	8 (9.5%)	
Proficient	261 (91.9%)	185 (92.5%)	76 (90.5%)	
EBV				0.0131
Positive	22 (7.7%)	10 (5.0%)	12 (14.3%)	
Negative	262 (92.3%)	190 (95.0%)	72 (85.7%)	
HER2				> 0.9999
Positive	31 (10.9%)	22 (11.0%)	9 (10.7%)	
Negative	253 (89.1%)	178 (89.0%)	75 (89.3%)	
PD-L1 (CPS ≥ 1)				0.2564
Positive	245 (86.3%)	169 (84.5%)	76 (90.5%)	
Negative	39 (13.7%)	31 (15.5%)	8 (9.5%)	
PD-L1 (CPS ≥ 5)				0.0334
Positive	111 (39.1%)	70 (35.0%)	41 (48.8%)	
Negative	173 (60.9%)	130 (65.0%)	43 (51.2%)	

Data are presented as number (%) unless otherwise indicated. CLDN18.2; claudin-18 isoform 2, CPS; combined positive score, EBV; Epstein-Barr virus, GC; gastric cancer, HER2; human epidermal growth factor receptor 2, MMR; mismatch repair, PD-L1; programmed cell death ligand 1, pTNM; pathological tumor-node-metastasis.

**Figure 2:**
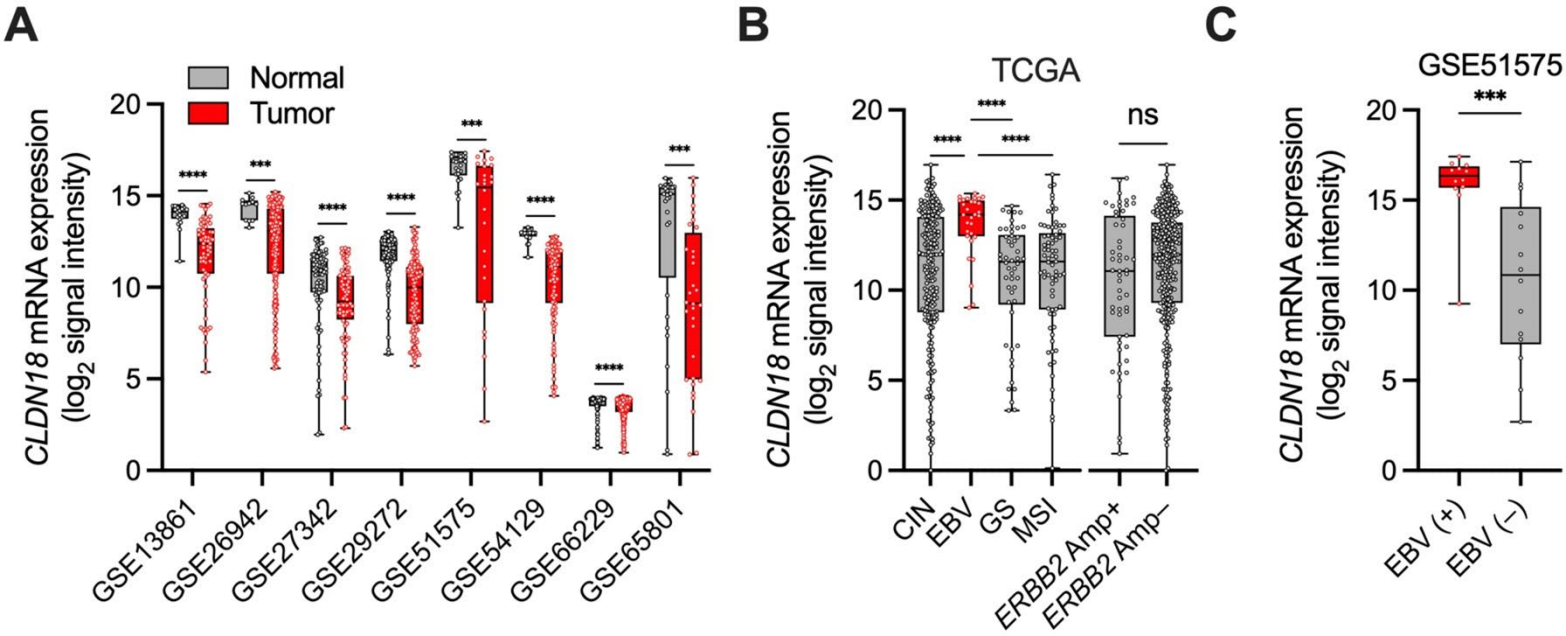
Association of *CLDN18* mRNA expression with molecular subtypes in GC (GC cohorts from TCGA and GEO). (**A**) mRNA expression of *CLDN18* in GC samples and paired normal tissues across eight cohorts from GEO (GSE13861: Normal, *n* = 19 vs Tumor, *n* = 65; GSE26942: Normal, *n* = 12 vs Tumor, *n* = 202; GSE27342: Normal, *n* = 80 vs Tumor, *n* = 80; GSE29272: Normal, *n* = 134 vs Tumor, *n* = 134; GSE51575: Normal, *n* = 26 vs Tumor, *n* = 26; GSE54129: Normal, *n* = 21 vs Tumor, *n* = 111; GSE66229: Normal, *n* = 100 vs Tumor, *n* = 300; GSE65801: Normal, *n* = 32 vs Tumor, *n* = 32). (**B**) mRNA expression of *CLDN18* in each molecular subtype of GC in TCGA cohort (CIN, *n* = 221; EBV, *n* = 30; GS, *n* = 50; MSI, *n* = 73; ERBB2 Amp + , *n* = 55; *ERBB2* Amp–, *n* = 352). (**C**) mRNA expression of *CLDN18* between EBV ( +) (*n* = 12) and EBV (–) (*n* = 14) GCs in GSE51575. Statistical significance was determined by the Mann–Whitney *U* test (**A**–**C**) and the Kruskal–Wallis test with Dunn’s multiple comparisons test (**B**). ***p < 0.001, ****p < 0.0001.

Given that CLDN18.2 represents one of the splicing forms of *CLDN18*, we examined mRNA expression pattern of *CLDN18* in GC by analyzing public datasets from TCGA and GEO. *CLDN18* mRNA expression was significantly decreased in GC tissues compared to adjacent normal mucosa (Fig. 2A). Among four subtypes of GC [microsatellite instability (MSI), EBV, genomically stable (GS), and chromosomal instability (CIN)] and HER2, *CLDN18* expression was significantly and distinctly higher in EBV-associated GC [EBV ( +)], while *ERBB2* (HER2) status did not influence CLDN18.2 expression (Fig. 2B). Furthermore, higher expression of *CLDN18* in EBV ( +) GC compared to EBV (–) GC was also observed in GSE51575 (Fig. 2C). These results suggest that the expression of CLDN18.2 is highly maintained in EBV ( +) GC compared to other molecular subtypes of GC, possibly due to the elevated expression of *CLDN18* mRNA.

### Impact of CLDN18.2 expression on tumor-infiltrating NK cells and macrophages in GC patients

**Figure 3:**
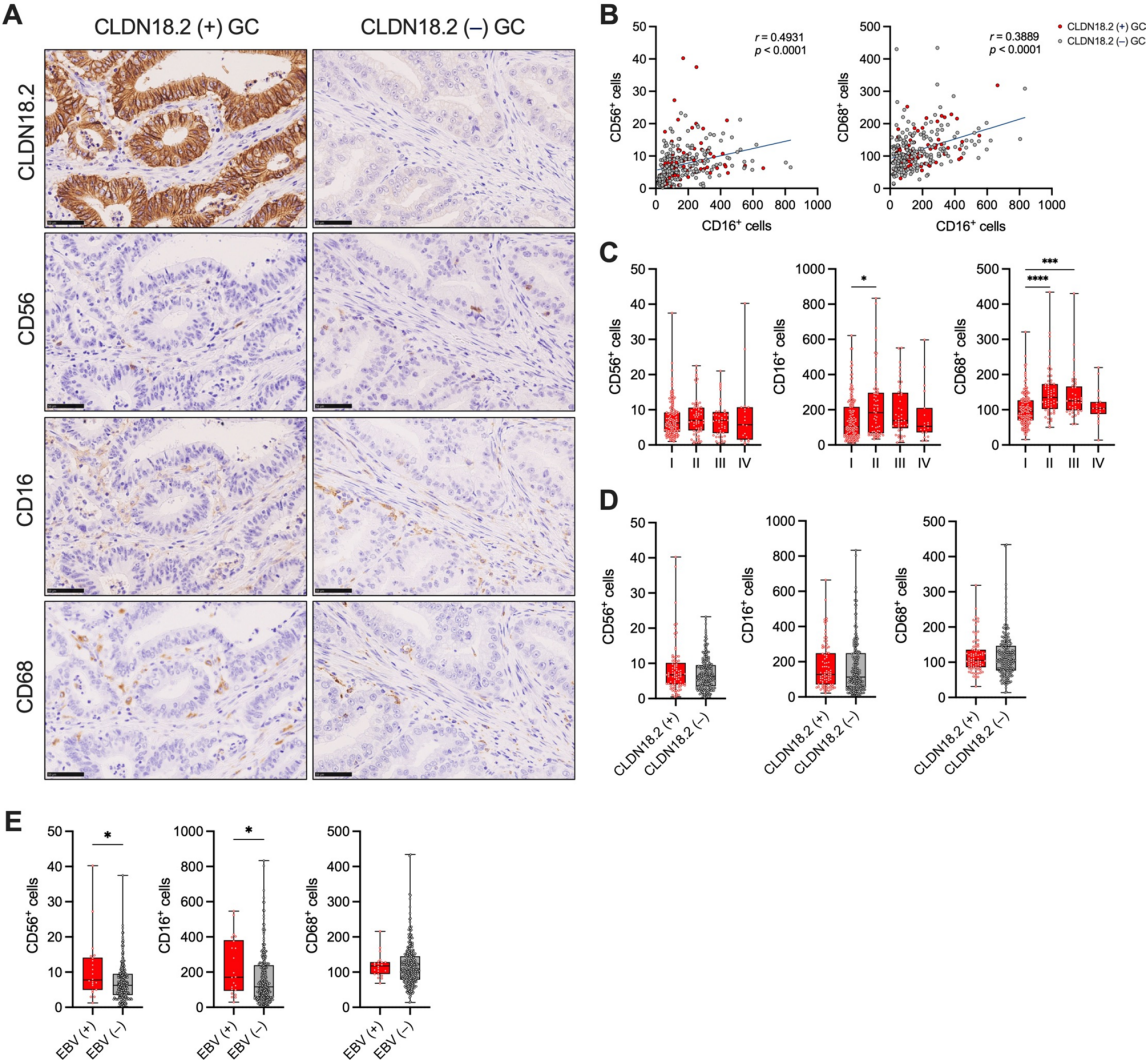
Association of CLDN18.2 expression with the infiltration of NK cells and macrophages in GC (FMU cohort 1). (**A**) Representative IHC images for CLDN18.2, CD56, CD16, and CD68 in CLDN18.2 ( +) and CLDN18.2 (–) GCs in FMU cohort 1 (*n* = 284). Scale bars: 50 μm. (**B**) Correlations between the number of tumor-infiltrating CD16^+^ cells and CD56^+^ cells or CD68^+^ cells detected by IHC in GCs (*n* = 284). (**C**) Comparison of the number of CD56^+^ cells, CD16^+^ cells, and CD68^+^ cells detected by IHC across all pathological TNM stages (I, *n* = 159; II, *n* = 61; III, *n* = 45; IV, *n* = 19) in GC. (**D**) Associations between CLDN18.2 expression and infiltration of CD56^+^ cells, CD16^+^ cells, or CD68^+^ cells detected by IHC in GC [CLDN18.2 ( +), *n* = 84; CLDN18.2 (–), *n* = 200]. (**E**) Associations between EBV status and infiltration of CD56^+^ cells, CD16^+^ cells, or CD68^+^ cells detected by in situ hybridization (for EBV) or IHC in GC [EBV ( +), *n* = 22; EBV (–), *n* = 262]. Statistical significance was determined by the Spearman rank-correlation coefficient (**B**), the Kruskal–Wallis test with Dunn’s multiple comparisons test (**C**), and the Mann–Whitney *U* test (**D**,**E**). *p < 0.05, ***p < 0.001, ****p < 0.0001.

We subsequently assessed the association between CLDN18.2 expression and the infiltration of NK cells and macrophages in GC. CD56 and CD68, as well as CD16, served as markers for NK cells and macrophages, respectively (Fig. 3A). The number of CD16^+^ cells significantly correlated with the number of CD56^+^ cells and CD68^+^ cells (Fig. 3B), suggesting that CD16 might be expressed in both NK cells and macrophages. The number of CD56^+^ cells was comparable across all pTNM stages in GC (Fig. 3C), whereas the numbers of CD68^+^ cells and CD16^+^ cells, were significantly higher in pTNM stage II or III GCs than those in pTNM stage I GC (Fig. 3C). Moreover, no significant associations between CLDN18.2 expression and the numbers of CD56^+^ cells, CD16^+^ cells, or CD68^+^ cells were observed in GC (Fig. 3D), suggesting that CLDN18.2 expression did not affect the infiltration of NK cells and macrophages in GC. We also investigated the associations between CLDN18.2 expression and the frequencies of CD56^dim^CD16^+^ or CD56^bright^CD16^−^ NK cells in GC. Fifteen GC patients were enrolled for this purpose (FMU cohort 2, Supplementary Table S1). The percentages of CD56^dim^CD16^+^ or CD56^bright^CD16^−^ NK cells did not differ across all pTNM stages in GC (Supplementary Fig. S1A). Furthermore, the frequencies of CD56^bright^CD16^−^ and CD56^dim^CD16^+^ NK cells were comparable between CLDN18.2 ( +) and CLDN18.2 (–) GCs (Supplementary Fig. S1B).

We further investigated the association of the infiltrations of NK cells and macrophages with EBV status in GC because the frequency of CLDN18.2 ( +) cases was significantly high in EBV ( +) GC (Fig. 1E). The numbers of CD56^+^ cells and CD16^+^ cells were significantly higher in EBV ( +) GC compared to EBV (–) GC, whereas the number of CD68^+^ cells was comparable between EBV ( +) and EBV (–) GCs (Fig. 3E). Furthermore, the analysis of datasets from TCGA and GEO revealed that the NK cell gene signature showed significantly higher expression in the EBV ( +) GC subtype compared to the other three subtypes (Supplementary Fig. S2A). This higher expression of NK cell gene signature in EBV ( +) GC compared to EBV (–) GC was also observed in GSE51575 (Supplementary Fig. S2B). These results suggest that both CLDN18.2 expression and the number of tumor-infiltrating NK cells were significantly higher in EBV ( +) GC compared to other molecular subtypes.

### Impact of CLDN18.2 expression on peripheral blood NK cells and monocytes in GC patients

**Figure 4:**
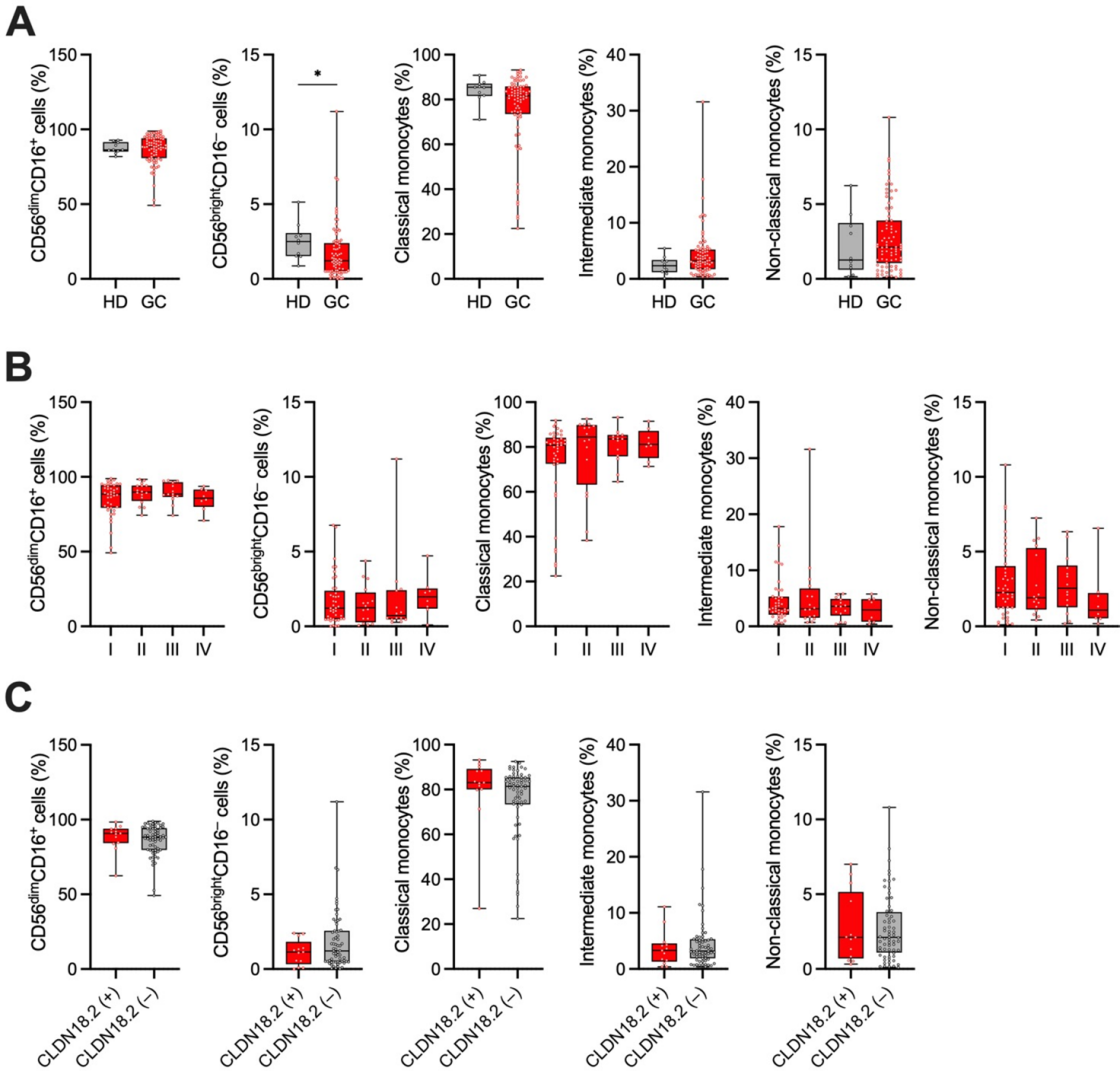
Association of CLDN18.2 expression with the frequencies of peripheral blood NK cells and monocytes in GC (FMU cohort 3). (**A**) Comparison of the frequencies of CD56^dim^CD16^+^ NK cells, CD56^bright^CD16^−^ NK cells, classical monocytes, intermediate monocytes, and nonclassical monocytes in PBMCs detected by flow cytometry between healthy donors (HD) (*n* = 10) and GC patients (GC) (*n* = 79). (**B**) Comparison of the frequencies of the CD56^dim^CD16^+^ NK cells, CD56^bright^CD16^−^ NK cells, classical monocytes, intermediate monocytes, and nonclassical monocytes in PBMCs detected by flow cytometry across all pathological TNM stages (I, *n* = 41; II, *n* = 16; III, *n* = 14; IV, *n* = 8) in patients with GC. (**C**) Associations between CLDN18.2 expression and the frequencies of CD56^dim^CD16^+^ NK cells, CD56^bright^CD16^−^ NK cells, classical monocytes, intermediate monocytes, and nonclassical monocytes in PBMCs in patients with GC [CLDN18.2 ( +), *n* = 13; CLDN18.2 (–), *n* = 64]. The frequency of each subset of NK cells and monocytes was determined by flow cytometry, while CLDN18.2 positivity was assessed by IHC using surgically resected GC specimens. Statistical significance was determined by the the Mann–Whitney *U* test (**A**,**C**) and the Kruskal–Wallis test with Dunn’s multiple comparisons test (**B**). *p < 0.05.

Peripheral blood NK cells and monocytes are important sources of tumor-infiltrating NK cells and monocytes/macropahges. Thus, we finally examined the association between CLDN18.2 expression in GC tumor tissues and the frequency of peripheral blood NK cells, including CD56^dim^CD16^+^ and CD56^bright^CD16^−^ NK cells, as well as monocytes, including classical (CD14^++^CD16^–^), intermediate (CD14^++^CD16^+^), and non-classical monocytes (CD14^+^CD16^++^) (Supplementary Fig. S3). We enrolled 79 patients with GC for this analysis (FMU cohort 3, Table 2). The frequency of CD56^bright^CD16^−^ NK cells was significantly decreased in the GC patients compared to healthy donors (HDs) (Fig. 4A), while the frequencies of other subsets of NK cells and monocytes were comparable between the GC patients and HDs (Fig. 4A). Moreover, the frequencies of all subsets of NK cells and monocytes did not differ across all pTNM stages in GC (Fig. 4B). Furthermore, there were no significant associations between CLDN18.2 expression in tumor tissues and the frequency of all subsets of NK cells and monocytes in the GC patients (Fig. 4C), suggesting that CLDN18.2 expression might not affect the frequency of peripheral blood NK cells and monocytes in GC.

**Table 2:** Clinicopathological characteristics of gastric cancer patients (FMU cohort 3).

	Total
	*n* = 79
Age	
Mean ± SD	69.7 ± 11.7
Gender	
Male	47 (59.5%)
Female	32 (40.5%)
Location	
U	19 (24.1%)
M	21 (26.6%)
L	37 (46.8%)
Remnant GC	2 (2.5%)
Histological type	
Differentiated	33 (41.8%)
Undifferentiated	45 (56.9%)
Unclear	1 (1.3%)
Tumor invasion	
T1	35 (44.3%)
T2	13 (16.4%)
T3	7 (8.9%)
T4	24 (30.4%)
Lymph node metastasis	
Absent	48 (60.8%)
Present	31 (39.2%)
Distant metastasis	
Absent	71 (89.9%)
Present	8 (10.1%)
pTNM stage	
I	41 (51.9%)
II	16 (20.3%)
III	14 (17.7%)
IV	8 (10.1%)
CLDN18.2	
Positive	13 (16.5%)
Negative	64 (81.0%)
Not available	2 (2.5%)

Data are presented as number (%) unless otherwise indicated.

CLDN18.2; claudin-18 isoform 2, GC; gastric cancer, pTNM; pathological tumor-node-metastasis.

## Discussion

In the present study, we investigated the impact of CLDN18.2 expression on tumor-infiltrating NK cells and macrophages, as well as peripheral blood NK cells and monocytes, in patients with GC. The numbers of tumor-infiltrating NK cells and macrophages, as well as the frequencies of peripheral blood NK cells and monocytes, were comparable between CLDN18.2 ( +) and CLDN18.2 (–) GCs, suggesting that CLDN18.2 expression has no significant adverse impacts on the ADCC-related compornents, particularly the frequency of NK cells and monocytes/macrophages, in GC patients. Additionally, CLDN18.2 expression and NK cell infiltration were significantly higher in EBV ( +) GC compared to other molecular subtypes. These results suggest that EBV ( +) GC, particularly CLDN18.2 ( +) cases accounting for more than 50% of all EBV ( +) cases, might be a suitable target for zolbetuximab therapy.

Our current results suggest that CLDN18.2 expression has no significant impact on prognosis in GC patients (Fig. 1C). Consensus regarding the impact of CLDN18.2 expression on clinical outcomes in GC patients is still lacking. Jun et al*.* and Sanada et al*.* reported that loss or downregulation of CLDN18.2 expression was associated with poor survival in patients with GC^26,27^. Jun et al. found that decreased expression of CLDN18.2 was an independent indicator of poor prognosis in GC patients^26^. On the other hand, Wang et al*.* and Jia et al*.* reported that positive expression of CLDN18.2 was significantly associated with poor OS in GC patients^28,29^. Wang et al*.* identified CLDN18.2 expression as an independent risk factor for patient prognosis (*p* = 0.003, hazard ratio = 1.37, 95% CI 1.03–1.81)^28^. Furthermore, Kubota et al*.* and Kayikcioglu et al*.* reported that there were no significant associations between CLDN18.2 expression and prognosis, including progression-free survival and OS, in patients with unresectable locally advanced or metastatic GC^30,31^. CLDN18.2 has been reported to be involved in maintaining the barrier function of gastric mucosal cells to prevent leakage of H^+^ and other cations^32^. Downregulation of CLDN18.2 expression, which is observed in 57.5% of GC cases^27^, disrupts the homeostasis of gastric mucosal cells, and may be involved in the early stages of GC development^27,33^. However, the remaining CLDN18.2 becomes highly and stably expressed in specific tumor tissues, probably contributing to the proliferation and differentiation of tumor cells^34^. Furthermore, the results of the current study indicate that the frequency of CDLN18.2 ( +) cases was the lowest in pTNM stage I GC, and gradually increased depending on the pTNM staging. To assess the actual prognostic impact of CLDN18.2 in GC patients, a meta-analysis using comprehensive adjusted outcome data in each pTNM stage may be needed.

The frequency of CLDN18.2 ( +) cases was significantly higher in EBV ( +) GC compared to EBV (–) GC (Fig. 1E). Similar trends were also observed in previous studies by Coati et al*.*^35^ and Pellino et al*.*^36^. Based on our current analyses using public datasets of GC, mRNA expression of *CLDN18* was maintained at a high level in EBV ( +) GC (Figs. 2B and 2C), suggesting that CLDN18.2 might remain high in EBV ( +) GC. Yano et al*.* demonstrated that the extracellular signal-regulated kinase (ERK)/mitogen-activated protein kinase (MAPK) pathway was involved in upregulating mRNA expression of *CLDN18* in GC cells^37^. Additionally, the ERK/MAPK pathway has been reported to be constantly activated by the latent membrane protein 2A (LMP2A, an EBV protein) in GC cells^38^, which is expressed in approximately 50% of EBV ( +) GC^39^. Therefore, activation of the ERK/MAPK pathway induced by the LMP2A might be involved in the high expression of *CLDN18* mRNA and CLDN18.2 protein in EBV ( +) GC.

Immune checkpoint inhibitors (ICIs), including anti-programmed cell death 1 (PD-1)/PD-L1 antibodies, have been developed as a first-line chemotherapy in metastatic GC patients with tumors with a PD-L1 CPS of ≥ 1^40^. Furthermore, several combination therapies involving ICIs with chemotherapy, other molecular targeted therapy, or oligo-fractionated irradiation, have resulted in improved outcomes for patients with GC^41,42^. However, only a minority of GC patients benefit from ICI therapy and its combination therapies. Therefore, additional molecular targets for combination therapy with ICI are needed in the treatment of GC. In the present study, we found that CLDN18.2 status was significantly associated with PD-L1 expression (CPS ≥ 5) in GC patients (Table 1 and Fig. 1E). This result suggests that the combination therapy of zolbetuximab with anti-PD-1/PD-L1 antibodies may be a novel therapeutic strategy for CLDN18.2 ( +) GC. Elucidating the potential of combination therapy using anti-PD-1/PD-L1 antibodies and zolbetuximab in CLDN18.2 ( +) GC is the next line of our investigation.

In conclusion, CLDN18.2 expression has shown no significant adverse impacts on the frequencies of effector cells mediating ADCC, such as tumor-infiltrating NK cells and macrophages, as well as all subsets of peripheral blood NK cells and monocytes, in patients with GC. Additionally, the findings of the current study provide a novel insight into CLDN18.2-targeted therapy in CLDN18.2 ( +) GC, highlighting its effectiveness for EBV ( +) cases and its potential for combination therapy with anti-PD-1/PD-L1 antibodies.

## Methods

### Patient samples

We enrolled patients with GC who had undergone surgical resection at FMU hospital during different time periods: FMU cohort 1 (2013–2019; *n* = 284) (Figs. 1 and 3, Table 1), FMU cohort 2 (2023; *n* = 15) (Supplementary Fig. S1, Supplementary Table S1), and FMU cohort 3 (2019–2021; *n* = 79) (Fig. 4, Table 2). Clinical and pathological information was retrospectively collected by reviewing medical records. This study was approved by the Institutional Ethical Committee of FMU (Reference Nos. 2329 and REC2023-144), and all procedures were conducted in accordance with the Helsinki Declaration. Written informed consent to be included in the study was obtained from all participants.

### IHC analysis

Paraffin-embedded 4-μm sections of GC tissue were deparaffinized in xylene and rehydrated in ethanol. Endogenous peroxidases were blocked with 0.3% hydrogen peroxide in methanol. Antigens were retrieved by autoclaving with Target Retrieval Solution at pH 6.0 or pH 9.0 (Dako/Agilent Technologies, Santa Clara, CA). After washing with PBS, the sections were incubated overnight at 4 °C with primary antibodies (see Supplementary Table S2). Subsequently, the sections were incubated with HRP-conjugated anti-mouse or anti-rabbit secondary antibodies (K4003 or K4001, Dako/Agilent Technologies). Peroxidase activity was visualized with diaminobenzidine, and nuclei were counterstained with Mayer’s hematoxylin solution.

### Assessment of IHC

CLDN18.2 positivity was defined as ≥ 75% of tumor cells exhibiting moderate-to-strong membranous CLDN18 staining (Supplementary Fig. S4), as previously described^30^. CLDN18.2 positivity in GC patients from FMU cohorts 1–3 was determined by IHC analysis of surgically resected GC specimens. To assess CD16^+^, CD56^+^, and CD68^+^ cells, we reviewed four independent areas in the tumor core of GC tissues, counting the number of cells at a magnification of × 400. IHC analyses of MMR, HER2, and PD-L1 CPS, and an in situ hybridization analysis of the integrated EBV genome were performed as previously reported^43–45^. The IHC evaluation was conducted by at least two observers (A.M and S.N) who were blinded to all clinical and pathological information.

### Dissociation of human GC tissues

Resected GC samples were minced into small pieces and digested using the gentleMACS™ Octo Dissociator with Heaters (#130–096-427, Miltenyi Biotec, Bergisch Gladbach, Germany) and the Tumor Dissociation Kit, human (#130–095-929, Miltenyi Biotec) following the manufacturer’s protocol. After completing the gentleMACS Dissociator program, the cell suspension was passed through a 70-μm strainer and centrifuged. The collected cells were then washed twice with RPMI and utilized for further experiments.

### Isolation of peripheral blood mononuclear cells

Peripheral blood mononuclear cells (PBMCs) were isolated from GC patients (*n* = 79, FMU cohort 3) and HD (*n* = 10) using the BD Vacutainer® CPT™ system (362,753, BD Biosciences, San Jose, CA). Contaminating red blood cells were lysed by the BD Pharm Lyse™ (#555,899, BD Biosciences). After lysing red blood cells, the collected PBMCs were washed twice with PBS and employed for subsequent experiments.

### Flow cytometry analysis

PBMCs and cells isolated from GC tissues were incubated with primary antibodies (see Supplementary Table S2) for 30 min at 4 °C in the dark. Following PBS washing, the stained cells were incubated with 7-amino-actinomycin D (420,404, BioLegend, San Diego, CA) for 15 min at room temperature in the dark and then analyzed using a BD FACSCanto II flow cytometer (BD Biosciences). Flow cytometry data were analyzed with FlowJo software version 10.7.1 (FlowJo, Ashland, OR, proprietary commercial software). The gating strategy employed in this study is illustrated in Supplementary Fig. S3.

### Data analyses of public datasets

We obtained publicly accessible datasets of mRNA expression in stomach adenocarcinoma from TCGA via cBioPortal (https://www.cbioportal.org)^46^ and GEO. For comparison of *CLDN18* mRNA expression levels, we compared GC tissues with adjacent normal mucosa, EBV ( +) versus EBV (–), HER2 ( +) versus HER2 (–), and across four molecular subtypes of GCs (MSI, EBV, GS, and CIN). The log_2_ signal intensity of *CLDN18* was extracted from TCGA-STAD, GSE13861, GSE26942, GSE27342, GSE29272, GSE51575, GSE54129, GSE66229, and GSE65801. Additionally, we calculated a multi-gene expression signature for NK cells (*NKG7*, *GZMA*, *EOMES*, *SMAD3*, *TBX21*, *KLRK1*, *NCR1*, *PLCG2*, *STYK1*) based on a previous report by Davoli et al.^47^. We compared these signature scores among four molecular subtypes of GCs and between EBV-associated [EBV ( +)] and EBV-negative [EBV (–)] GCs using TCGA and GSE51575.

### Statistical analysis

Data are presented as means ± SD. Statistical analyses were conducted using Graph Pad Prism 9 version 9.5.1 (Graph Pad Software, La Jolla, CA). We conducted two-group comparisons of means using the Mann–Whitney *U* test. For comparisons involving multiple groups, we employed the Kruskal–Wallis test with Dunn’s multiple comparisons test. Correlation analysis was conducted using the Spearman rank-correlation coefficient. Proportions of groups in categorical variables were compared using Fisher’s exact test or the Chi-square test. Kaplan–Meier estimates were employed to assess the probability of RFS or OS among 284 GC patients (FMU cohort 1) until December 2023. Differences in survival rates and recurrence rates between the two groups were analyzed using the log-rank test. A *p*-value of less than 0.05 was considered statistically significant.

### Supplementary Information

Supplementary Information.

## Data Availability

The datasets generated and/or analyzed during this study are available from the corresponding author on reasonable request.

## References

[1] Smyth, E. C., Nilsson, M., Grabsch, H. I., van Grieken, N. C. & Lordick, F. Gastric cancer. *Lancet* **396**, 635–648. 10.1016/s0140-6736(20)31288-5 (2020). 32861308 10.1016/s0140-6736(20)31288-5

[2] Siegel, R. L., Miller, K. D., Fuchs, H. E. & Jemal, A. Cancer statistics, 2022. *CA Cancer J. Clin.* **72**, 7–33. 10.3322/caac.21708 (2022). 35020204 10.3322/caac.21708

[3] Sahin, U. *et al.* Claudin-18 splice variant 2 is a pan-cancer target suitable for therapeutic antibody development. *Clin. Cancer Res.* **14**, 7624–7634. 10.1158/1078-0432.Ccr-08-1547 (2008). 19047087 10.1158/1078-0432.Ccr-08-1547

[4] Sahin, U. *et al.* FAST: A randomised phase II study of zolbetuximab (IMAB362) plus EOX versus EOX alone for first-line treatment of advanced CLDN18.2-positive gastric and gastro-oesophageal adenocarcinoma. *Ann. Oncol.* **32**, 609–619. 10.1016/j.annonc.2021.02.005 (2021). 10.1016/j.annonc.2021.02.005 33610734

[5] Lordick, F. *et al.* Patient-reported outcomes from the phase II FAST trial of zolbetuximab plus EOX compared to EOX alone as first-line treatment of patients with metastatic CLDN18.2+ gastroesophageal adenocarcinoma. *Gastric Cancer* **24**, 721–730. 10.1007/s10120-020-01153-6 (2021). 10.1007/s10120-020-01153-6 PMC8064997 33755863

[6] Shitara, K. *et al.* Zolbetuximab plus mFOLFOX6 in patients with CLDN18.2-positive, HER2-negative, untreated, locally advanced unresectable or metastatic gastric or gastro-oesophageal junction adenocarcinoma (SPOTLIGHT): A multicentre, randomised, double-blind, phase 3 trial. *Lancet* **401**, 1655–1668. 10.1016/s0140-6736(23)00620-7 (2023). 10.1016/S0140-6736(23)00620-7 37068504

[7] Shah, M. A. *et al.* Zolbetuximab plus CAPOX in CLDN18.2-positive gastric or gastroesophageal junction adenocarcinoma: the randomized, phase 3 GLOW trial. *Nat. Med.* **29**, 2133–2141. 10.1038/s41591-023-02465-7 (2023). 10.1038/s41591-023-02465-7 PMC10427418 37524953

[8] Lordick, F. *et al.* Immunological effects and activity of multiple doses of zolbetuximab in combination with zoledronic acid and interleukin-2 in a phase 1 study in patients with advanced gastric and gastroesophageal junction cancer. *J. Cancer Res. Clin. Oncol.* **149**, 5937–5950. 10.1007/s00432-022-04459-3 (2023). 36607429 10.1007/s00432-022-04459-3 PMC10356865

[9] Lo Nigro, C. *et al.* NK-mediated antibody-dependent cell-mediated cytotoxicity in solid tumors: Biological evidence and clinical perspectives. *Ann. Transl. Med.* **7**, 105. 10.21037/atm.2019.01.42 (2019). 10.21037/atm.2019.01.42 PMC6462666 31019955

[10] Strowig, T., Brilot, F. & Münz, C. Noncytotoxic functions of NK cells: direct pathogen restriction and assistance to adaptive immunity. *J. Immunol.* **180**, 7785–7791. 10.4049/jimmunol.180.12.7785 (2008). 18523242 10.4049/jimmunol.180.12.7785 PMC2575662

[11] Izawa, S. *et al.* H₂O₂ production within tumor microenvironment inversely correlated with infiltration of CD56(dim) NK cells in gastric and esophageal cancer: Possible mechanisms of NK cell dysfunction. *Cancer Immunol. Immunother* **60**, 1801–1810. 10.1007/s00262-011-1082-7 (2011). 21811786 10.1007/s00262-011-1082-7 PMC11028881

[12] Saito, H., Takaya, S., Osaki, T. & Ikeguchi, M. Increased apoptosis and elevated Fas expression in circulating natural killer cells in gastric cancer patients. *Gastric Cancer* **16**, 473–479. 10.1007/s10120-012-0210-1 (2013). 23179366 10.1007/s10120-012-0210-1

[13] Li, T. *et al.* Gastric cancer cells inhibit natural killer cell proliferation and induce apoptosis via prostaglandin E2. *Oncoimmunology* **5**, e1069936. 10.1080/2162402x.2015.1069936 (2016). 27057432 10.1080/2162402x.2015.1069936 PMC4801461

[14] Wang, Z. *et al.* Activation of STAT3 in human gastric cancer cells via interleukin (IL)-6-type cytokine signaling correlates with clinical implications. *PLoS ONE* **8**, e75788. 10.1371/journal.pone.0075788 (2013). 24116074 10.1371/journal.pone.0075788 PMC3792128

[15] Wu, K. *et al.* Redefining tumor-associated macrophage subpopulations and functions in the tumor microenvironment. *Front. Immunol.* **11**, 1731. 10.3389/fimmu.2020.01731 (2020). 32849616 10.3389/fimmu.2020.01731 PMC7417513

[16] Chiang, C. F. *et al.* Metformin-treated cancer cells modulate macrophage polarization through AMPK-NF-κB signaling. *Oncotarget* **8**, 20706–20718. 10.18632/oncotarget.14982 (2017). 10.18632/oncotarget.14982 PMC5400538 28157701

[17] Chen, M. *et al.* Downregulation of triggering receptor expressed on myeloid cells 1 inhibits invasion and migration of liver cancer cells by mediating macrophage polarization. *Oncol Rep.*10.3892/or.2021.7988 (2021). 10.3892/or.2021.7988 33649843

[18] Helm, O. *et al.* Tumor-associated macrophages exhibit pro- and anti-inflammatory properties by which they impact on pancreatic tumorigenesis. *Int. J. Cancer* **135**, 843–861. 10.1002/ijc.28736 (2014). 24458546 10.1002/ijc.28736

[19] Pahl, J. H. *et al.* Macrophages inhibit human osteosarcoma cell growth after activation with the bacterial cell wall derivative liposomal muramyl tripeptide in combination with interferon-γ. *J. Exp. Clin. Cancer Res.* **33**, 27. 10.1186/1756-9966-33-27 (2014). 24612598 10.1186/1756-9966-33-27 PMC4007518

[20] van Ravenswaay Claasen, H. H., Kluin, P. M. & Fleuren, G. J. Tumor infiltrating cells in human cancer. On the possible role of CD16+ macrophages in antitumor cytotoxicity. *Lab. Investig.* **67**, 166–174. (1992). 1501443

[21] Zhu, Q., Wu, X., Tang, M. & Wu, L. Observation of tumor-associated macrophages expression in gastric cancer and its clinical pathological relationship. *Medicine (Baltimore)* **99**, e19839. 10.1097/md.0000000000019839 (2020). 10.1097/MD.0000000000019839 PMC7220635 32332633

[22] Stansfield, B. K. & Ingram, D. A. Clinical significance of monocyte heterogeneity. *Clin. Transl. Med.* **4**, 5. 10.1186/s40169-014-0040-3 (2015). 25852821 10.1186/s40169-014-0040-3 PMC4384980

[23] Yeap, W. H. *et al.* CD16 is indispensable for antibody-dependent cellular cytotoxicity by human monocytes. *Sci. Rep.* **6**, 34310. 10.1038/srep34310 (2016). 27670158 10.1038/srep34310 PMC5037471

[24] Eljaszewicz, A. J. *et al*. Gastric cancer increase the percentage of intermediate (CD14^++^CD16^+^) and nonclassical (CD14^+^CD16^+^) monocytes. *Cent. Eur. J. Immunol.* **37**, 355–361. 10.5114/ceji.2012.32725 (2012).

[25] Jeong, J. *et al.* Tumor-infiltrating neutrophils and non-classical monocytes may be potential therapeutic targets for HER2(negative) gastric cancer. *Immune Netw.* **21**, e31. 10.4110/in.2021.21.e31 (2021). 34522444 10.4110/in.2021.21.e31 PMC8410991

[26] Jun, K. H., Kim, J. H., Jung, J. H., Choi, H. J. & Chin, H. M. Expression of claudin-7 and loss of claudin-18 correlate with poor prognosis in gastric cancer. *Int. J. Surg.* **12**, 156–162. 10.1016/j.ijsu.2013.11.022 (2014). 24333468 10.1016/j.ijsu.2013.11.022

[27] Sanada, Y. *et al.* Down-regulation of the claudin-18 gene, identified through serial analysis of gene expression data analysis, in gastric cancer with an intestinal phenotype. *J. Pathol.* **208**, 633–642. 10.1002/path.1922 (2006). 16435283 10.1002/path.1922

[28] Wang, C. *et al.* CLDN18.2 expression and its impact on prognosis and the immune microenvironment in gastric cancer. *BMC Gastroenterol.* **23**, 283. 10.1186/s12876-023-02924-y (2023). 10.1186/s12876-023-02924-y PMC10428652 37582713

[29] Jia, K. *et al.* Multiplex immunohistochemistry defines the tumor immune microenvironment and immunotherapeutic outcome in CLDN18.2-positive gastric cancer. *BMC Med.* **20**, 223. 10.1186/s12916-022-02421-1 (2022). 10.1186/s12916-022-02421-1 PMC9272556 35811317

[30] Kubota, Y. *et al.* Comprehensive clinical and molecular characterization of claudin 18.2 expression in advanced gastric or gastroesophageal junction cancer. *ESMO Open* **8**, 100762. 10.1016/j.esmoop.2022.100762 (2023). 10.1016/j.esmoop.2022.100762 PMC10024138 36610262

[31] Kayikcioglu, E., Yüceer, R. O., Cetin, B., Yüceer, K. & Karahan, N. Prognostic value of claudin 18.2 expression in gastric adenocarcinoma. *World J. Gastrointest. Oncol.* **15**, 343–351. 10.4251/wjgo.v15.i2.343 (2023). 10.4251/wjgo.v15.i2.343 PMC9994048 36908327

[32] Hayashi, D. *et al.* Deficiency of claudin-18 causes paracellular H+ leakage, up-regulation of interleukin-1β, and atrophic gastritis in mice. *Gastroenterology* **142**, 292–304. 10.1053/j.gastro.2011.10.040 (2012). 22079592 10.1053/j.gastro.2011.10.040

[33] Oshima, T. *et al.* Down-regulation of claudin-18 is associated with the proliferative and invasive potential of gastric cancer at the invasive front. *PLoS ONE* **8**, e74757. 10.1371/journal.pone.0074757 (2013). 24073219 10.1371/journal.pone.0074757 PMC3779237

[34] Cao, W. *et al.* Claudin18.2 is a novel molecular biomarker for tumor-targeted immunotherapy. *Biomark. Res.* **10**, 38. 10.1186/s40364-022-00385-1 (2022). 10.1186/s40364-022-00385-1 PMC9153115 35642043

[35] Coati, I. *et al.* Claudin-18 expression in oesophagogastric adenocarcinomas: a tissue microarray study of 523 molecularly profiled cases. *Br. J. Cancer* **121**, 257–263. 10.1038/s41416-019-0508-4 (2019). 31235864 10.1038/s41416-019-0508-4 PMC6738069

[36] Pellino, A. *et al.* Association of CLDN18 protein expression with clinicopathological features and prognosis in advanced gastric and gastroesophageal junction adenocarcinomas. *J. Pers. Med.*10.3390/jpm11111095 (2021). 10.3390/jpm11111095 PMC8624955 34834447

[37] Yano, K., Imaeda, T. & Niimi, T. Transcriptional activation of the human claudin-18 gene promoter through two AP-1 motifs in PMA-stimulated MKN45 gastric cancer cells. *Am. J. Physiol. Gastrointest. Liver Physiol.* **294**, G336-343. 10.1152/ajpgi.00328.2007 (2008). 18032479 10.1152/ajpgi.00328.2007

[38] Iwakiri, D., Minamitani, T. & Samanta, M. Epstein-Barr virus latent membrane protein 2A contributes to anoikis resistance through ERK activation. *J. Virol.* **87**, 8227–8234. 10.1128/jvi.01089-13 (2013). 23698301 10.1128/jvi.01089-13 PMC3700196

[39] Imai, S. *et al.* Gastric carcinoma: monoclonal epithelial malignant cells expressing Epstein-Barr virus latent infection protein. *Proc. Natl. Acad. Sci. USA* **91**, 9131–9135. 10.1073/pnas.91.19.9131 (1994). 8090780 10.1073/pnas.91.19.9131 PMC44761

[40] Janjigian, Y. Y. *et al.* First-line nivolumab plus chemotherapy versus chemotherapy alone for advanced gastric, gastro-oesophageal junction, and oesophageal adenocarcinoma (CheckMate 649): A randomised, open-label, phase 3 trial. *Lancet* **398**, 27–40. 10.1016/s0140-6736(21)00797-2 (2021). 34102137 10.1016/s0140-6736(21)00797-2 PMC8436782

[41] Song, X. *et al.* Immune checkpoint inhibitor combination therapy for gastric cancer: Research progress. *Oncol. Lett.* **20**, 46. 10.3892/ol.2020.11905 (2020). 32802168 10.3892/ol.2020.11905 PMC7412728

[42] Mimura, K. *et al.* Combination of oligo-fractionated irradiation with nivolumab can induce immune modulation in gastric cancer. *J. Immunother. Cancer*. 10.1136/jitc-2023-008385 (2024). 10.1136/jitc-2023-008385 PMC10828861 38290769

[43] Noda, M. *et al.* Glycosyltransferase gene expression identifies a poor prognostic colorectal cancer subtype associated with mismatch repair deficiency and incomplete glycan synthesis. *Clin. Cancer Res.* **24**, 4468–4481. 10.1158/1078-0432.Ccr-17-3533 (2018). 29844132 10.1158/1078-0432.Ccr-17-3533

[44] Nakano, H. *et al.* PD-L1 overexpression in EBV-positive gastric cancer is caused by unique genomic or epigenomic mechanisms. *Sci. Rep.* **11**, 1982. 10.1038/s41598-021-81667-w (2021). 33479394 10.1038/s41598-021-81667-w PMC7820576

[45] Fukai, S. *et al.* Down-regulation of stimulator of interferon genes (STING) expression and CD8(+) T-cell infiltration depending on HER2 heterogeneity in HER2-positive gastric cancer. *Gastric Cancer* **26**, 878–890. 10.1007/s10120-023-01417-x (2023). 37542528 10.1007/s10120-023-01417-x

[46] Gao, J. *et al.* Integrative analysis of complex cancer genomics and clinical profiles using the cBioPortal. *Sci Signal* **6**, pl1. 10.1126/scisignal.2004088 (2013). 10.1126/scisignal.2004088 PMC4160307 23550210

[47] Davoli, T., Uno, H., Wooten, E. C. & Elledge, S. J. Tumor aneuploidy correlates with markers of immune evasion and with reduced response to immunotherapy. *Science*. 10.1126/science.aaf8399 (2017). 10.1126/science.aaf8399 PMC5592794 28104840

