# Supplementary material for: The impact of CLDN18.2 expression on effector cells mediating antibody-dependent cellular cytotoxicity in gastric cancer
Source: Sci Rep. 2024 Aug 2;14:17916. doi: 10.1038/s41598-024-68970-y (PMC11297210; doi:10.1038/s41598-024-68970-y)
Supplement: Supplementary file 1 — Supplementary Information. [file 41598_2024_68970_MOESM1_ESM.pdf]

The impact of CLDN18.2 expression on effector cells mediating antibody-dependent cellular cytotoxicity in gastric cancer

Authors: Akira Matsuishi,[1] Shotaro Nakajima,[1,2,†] Motonobu Saito,[1] Katsuharu Saito,[1] Satoshi Fukai,[1] Hideaki Tsumuraya,[1] Ryo Kanoda,[1] Tomohiro Kikuchi,[1] Azuma Nirei,[1] Akinao Kaneta,[1] Hirokazu Okayama,[1] Kosaku Mimura,[1,3] Hiroyuki Hanayama,[1] Wataru Sakamoto,[1] Tomoyuki Momma,[1] Zenichiro Saze,[1] and Koji Kono[1,2]

Authors' Affiliations: [1]Department of Gastrointestinal Tract Surgery, [2]Department of Multidisciplinary Treatment of Cancer and Regional Medical Support, [3]Department of Blood Transfusion and Transplantation Immunology, Fukushima Medical University School of Medicine, Fukushima, Japan

[†] **Corresponding Authors**: Shotaro Nakajima, Ph.D. Department of Multidisciplinary Treatment of Cancer and Regional Medical Support, Fukushima Medical University School of Medicine, 1 Hikariga-oka, Fukushima city, Fukushima 960-1295, Japan. Tel.:+81-24-547-1259; Fax.:+81-24-547-1980; E-mail: ipsho555@gmail.com

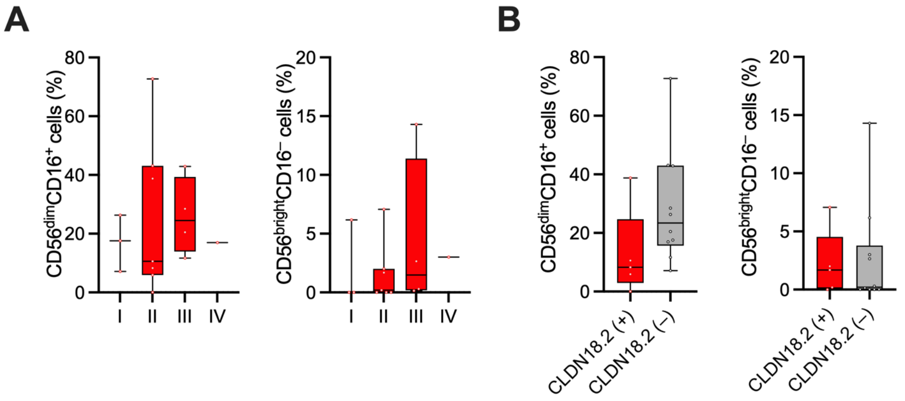

Supplementary Figure S1. Association between CLDN18.2 expression and the frequencies of tumor-infilatrating CD56^{bright}CD16$^{–}$ or CD56^{dim}CD16^{+} NK cells in GC (FMU cohort 2). (A) Comparison of the frequencies of tumor-infiltrating CD56^{bright}CD16$^{–}$ or CD56^{dim}CD16^{+} NK cells detected by flow cytometry of dissociated tissues across all TNM stages (I, *n* = 3; II, *n* = 7; III, *n* = 4; IV, *n* = 1) in GC. (B) Associations between CLDN18.2 expression and the frequencies of tumor-infiltrating CD56^{bright}CD16$^{–}$ or CD56^{dim}CD16^{+} NK cells in GC [CLDN18.2 (+), *n* = 5; CLDN18.2 (–), *n* = 10]. The frequency of each tumor-infiltrating NK cell subset was determined by flow cytometry of dissociated tissues, while CLDN18.2 positivity was assessed by IHC using surgically resected GC specimens. Statistical significance was determined by the Kruskal–Wallis test with the Dunn's multiple comparisons test (A) or the Mann–Whitney *U* test (B).

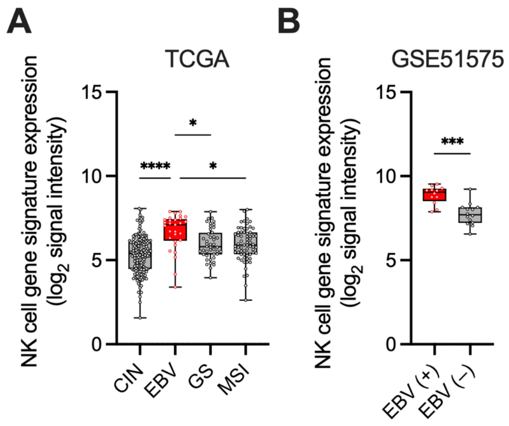

Supplementary Figure S2. Association between CLDN18 mRNA expression and molecular characteristics in GC (GC cohorts from TCGA and GEO). (**A**) Expressions of NK cell gene signature in each molecular subtype of GC (CIN, n = 179; EBV, n = 29; GS, n = 44; MSI, n = 67) in TCGA cohort. (**B**) Expression of NK cell gene signature in EBV (+) (n = 12) and EBV (–) (n = 14) GCs in GSE51575. Statistical significance was determined by the Kruskal-Wallis test with Dunn's multiple comparisons test (**A**), and the Mann-Whitney U test (**B**). *$p < 0.05$, ***$p < 0.001$, ****$p < 0.0001$.

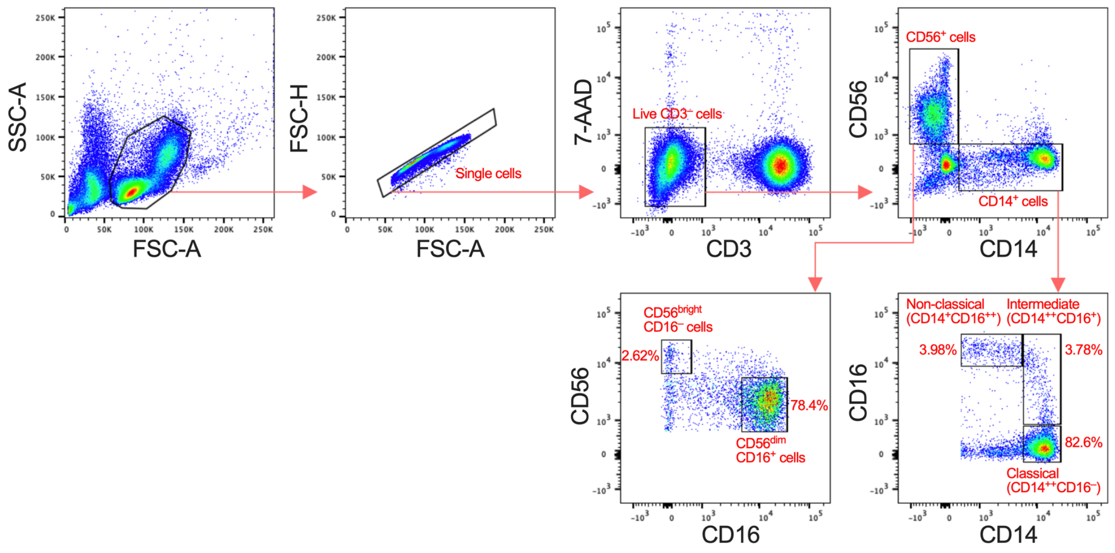

Supplementary Figure S3. Flow cytometry gating strategy used to define CD56^{bright}CD16$^{–}$ NK cells, CD56^{dim}CD16^{+} NK cells, classical (CD14^{++}CD16$^{–}$), intermediate (CD14^{++}CD16^{+}), and non-classical monocytes (CD14^{+}CD16^{++}) in PBMCs in FMU cohort 3 (*n* = 79) or cells isolated from GC tissues (only for NK cells) in FMU cohort 2 (*n* = 15).

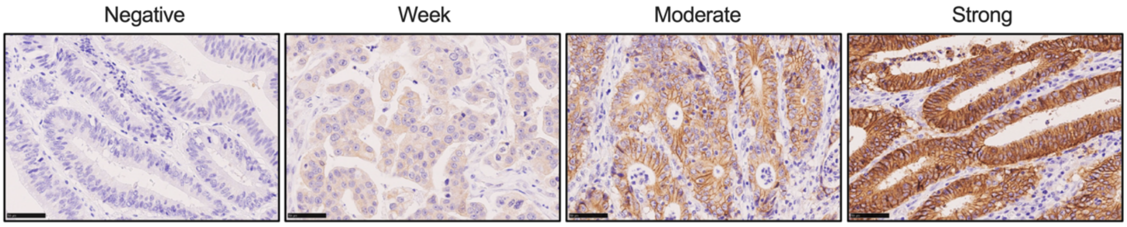

Supplementary Figure S4. Representative IHC images showing CLDN18.2 expression in GC across FMU cohorts 1–3.

Supplementary Table S1. Clinico-pathological characteristics in fifteen patients with GC (FMU cohort 2)

No.	Age	Gender	Location	Histological type	Tumor invasion	Lymph node metastasis	Distant metastasis	pTNM stage	CLDN18.2 status
1	68	Male	Remnant GC	Differentiated	pT3	pN0	Absent	ⅡA	Positive
2	63	Male	M	Unclear	pT2	pN1	Absent	ⅡA	Negative
3	68	Female	U	Undifferentiated	pT4a	pN2	Absent	ⅢA	Negative
4	79	Female	L	Differentiated	pT2	pN2	Absent	ⅡB	Positive
5	61	Male	U	Undifferentiated	pT2	pN2	Absent	ⅡB	Positive
6	62	Female	M	Undifferentiated	pT1a	pN0	Absent	ⅠA	Negative
7	62	Male	U	Differentiated	pT2	pN1	Absent	ⅡA	Negative
8	73	Male	L	Differentiated	pT2	pN0	Absent	ⅠB	Negative
9	64	Male	L	Undifferentiated	pT4a	pN0	Absent	ⅡB	Positive
10	76	Female	L	Undifferentiated	pT4a	pN3b	Present	Ⅳ	Negative
11	76	Male	U	Differentiated	pT4b	pN2	Absent	ⅢB	Negative
12	71	Male	L	Differentiated	pT2	pN1	Absent	ⅡA	Positive
13	72	Male	U	Undifferentiated	pT3	pN3a	Absent	ⅢB	Negative
14	76	Female	M	Undifferentiated	pT4a	pN3a	Absent	ⅢB	Negative
15	76	Male	L	Differentiated	pT1b2	pN0	Absent	ⅠA	Negative

CLDN18.2; claudin-18 isoform 2, pTNM; pathological tumor-node-metastasis

Supplementary Table S2. A list of antibodies used in this study

Reagent	Source	Identifier	Dilution
**IHC**			
Claudin 18 Monoclonal antibody	proteintech	Cat# 66167-1-Ig, AB_2881563	1:1000
CD16 (2H7) Mouse mAb	Cell Signaling Technology	Cat# 88251, AB_3076593	1:500
NCAM1 (CD56) (E7X9M) XP Rabbit mAb	Cell Signaling Technology	Cat# 99746, AB_2868490	1:300
HER2/ErbB2 (D8F12) XP Rabbit mAb	Cell Signaling Technology	Cat# 4290, AB_10557104	1:500
PD-L1 (E1L3N) XP Rabbit mAb	Cell Signaling Technology	Cat# 13684, AB_2687655	1:400
Monoclonal Mouse Anti-Human CD68	Dako/Agilent	Cat# M0876, AB_2074844	1:100
Monoclonal Mouse Anti-Human MLH 1	Dako/Agilent	Cat# M3640	1:50
Monoclonal Mouse Anti-Human MSH 2	Dako/Agilent	Cat# M3639	1:50
Monoclonal Rabbit Anti-Human MSH 6	Dako/Agilent	Cat# M3646	1:200
Monoclonal Rabbit Anti-Human PMS2	Dako/Agilent	Cat# M3647	1:50
**Flow cytometry**			
Brilliant Violet 421™ anti-human CD3	BioLegend	Cat# 300434, AB_10962690	1:50
APC/Cyanine7 anti-human CD14	BioLegend	Cat# 325620, AB_830693	1:50
PE/Cyanine7 anti-human CD16	BioLegend	Cat# 302016, AB_314215	1:50
FITC anti-human CD56 (NCAM)	BioLegend	Cat# 318304, AB_604100	1:50
BD Horizon™ BB515 Mouse Anti-Human CD56	BD Biosciences	Cat# 564488, AB_2744428	1:50

HER2; human epidermal growth factor receptor 2, IHC; immunohistochemistry, MLH1; MutL Protein Homolog 1, MSH2; MutS Protein Homolog 2, MSH6; MutS Protein Homolog 6, NCAM1; neural cell adhesion molecule 1, PD-L1; programmed cell death ligand 1,PMS2; Postmeiotic Segregation Increased 2
